# Soybean Plant Metabolism under Water Deficit and Xenobiotic and Antioxidant Agent Application

**DOI:** 10.3390/biology9090266

**Published:** 2020-09-03

**Authors:** Julia Renata Schneider, Mariele Müller, Vilson Antonio Klein, Luciana Grazziotin Rossato-Grando, Rômulo Pillon Barcelos, Genei Antonio Dalmago, Geraldo Chavarria

**Affiliations:** 1 Plant Physiology Laboratory, Agronomy Post-Graduate Program, Faculty of Agronomy and Veterinary Medicine, Passo Fundo University, BR 285, Passo Fundo 99052-900, Rio Grande do Sul, Brazil; juliaschneider07@hotmail.com (J.R.S.); muller.mariele@yahoo.com.br (M.M.); 2 Soil Physics Laboratory, Agronomy Post-Graduate Programa, Faculty of Agronomy and Veterinary Medicine, Passo Fundo University, BR 285, Passo Fundo 99052-900, Rio Grande do Sul, Brazil; vaklein@upf.br; 3 Faculty of Pharmacy, Institute of Biological Sciences, Bioexperimentation Post-Graduate Program, Passo Fundo University, BR 285, Passo Fundo 99052-900, Rio Grande do Sul, Brazil; rossatoluciana@upf.br (L.G.R.-G.); romulopillon@upf.br (R.P.B.); 4 Sustainable Production Systems, Ecophysiology, Embrapa Wheat, Rodovia BR 285, Km 294, Passo Fundo 99050-970, Rio Grande do Sul, Brazil; genei.dalmago@embrapa.br

**Keywords:** oxidative stress, oxidative damages, physiology, biochemistry, antioxidant defense, soil water potential, biostimulant

## Abstract

The aim was to evaluate the interactive effects on biochemistry and physiology of soybean plants exposed to simultaneous xenobiotic and water deficit stresses, and the possible attenuation of plant damage by an antioxidant agent. Soybean plants were submitted to eight different soil water potentials, in two experiments (first experiment: −0.96, −0.38, −0.07, −0.02 MPa, and second experiment: −3.09, −1.38, −0.69, −0.14 MPa), xenobiotic, and antioxidant agent applications. Was observed a reduction in water status, gas exchange, photosynthetic pigments, photosystem II quantum yield, and increased leaf temperature in plants under low water availability. Water deficit also induced oxidative stress by the increased production of reactive oxygen species, cellular and molecular damage, and induction of the antioxidant defense metabolism, reduction of gas exchange, water status, and photosynthetic efficiency. The xenobiotic application also caused changes, with deleterious effects more pronounced in low soil water availability, mainly the reactive oxygen species production, consequently the antioxidant activity, and the oxidative damages. This indicates different responses to the combination of stresses. Antioxidant enzyme activity was reduced by the application of the antioxidant agent. Principal Component Analysis showed a relation with the antioxidant agent and reactive oxygen species, which is probably due to signaling function, and with defense antioxidant system, mainly glutathione, represented by thiols.

## 1. Introduction

Plants are constantly exposed to environmental stresses responsible for physiological changes and oxidative stress. Water deficit stress is the major stressor and presents great potential to adversely affect crop development [1,2,3,4].

Sometimes, even under water deficit conditions, it is necessary to apply a fungicide. It occurs because most growers have been using the application time following one predetermined calendar schedule, according to the critical development stage of the plant, the first application, or even the residual period of the products [5]. This can lead plants to combined stress factors if the fungicide is a xenobiotic, which causes alterations in plant metabolism [6].

A large amount of research has shown plant responses to isolated stresses, which is incompatible with the reality of field conditions [7], but recent studies have confirmed that plants are exposed to a combination of stresses [7,8], therefore demanding findings in this sense. Thus, although plant performance in a water-restricted environment is already consolidated, still needs to elucidate plant to multiple stresses [9,10], such as water deficit stress and fungicide application.

In recent years, efforts have been made to understand plant responses to combined stresses [8,11]. A combined stress situation is when there is simultaneous exposure of the plant to different stress conditions, and means a new state of stress, and therefore requires new mechanisms and different pathways of defense response [7,10,11,12].

One situation of multiple stress factors that has been receiving attention in studies with is simultaneous water deficit and high temperature, which are almost dependent as it usually occurs at the same time in the field [13]. In these conditions, soybean was affected with greater intensity when water stress was imposed at higher temperatures [14]. The response is not a simple additive of every single stress, but a synergistic result of the combination [15]. In addition to these studies, soybean crop under water deficit stress is also evaluated recently in combination with UV-B radiation [16], cadmium [17], and heavy metal [18].

Antioxidant agents, also called biostimulants, are products used to minimize stress damage when plants are exposed to multiple stressors [19,20,21]. Any protective effects of these compounds against biotic and abiotic stresses would be associated with reductions in reactive oxygen species (ROS) and antioxidant system activation and increases in the production of phenolic compounds [20]. Thus, these products may act as osmoregulators, signaling molecules, and stomatal aperture modulators [22].

We hypothesize that soybean plants exposed to combined water deficit and fungicide application will have their metabolism more impaired than plants exposed to one stress type, or without stress. However, it is expected that stress damages will be minimized by the antioxidant agent application. Thus, the objective of this study is to evaluate the soybean plant metabolism under water deficit stress and the application of fungicide and antioxidant agents.

## 2. Materials and Methods

### 2.1. Plant Materials and Experimental Design

Soybean seeds of cultivar Intacta RR2PRO 5958 were seeded in 11 L plastic pots containing soil and substrate in a ratio of 2:1 and maintained four seedlings per pot. These were maintained in a growth chamber under conditions of 14 h of light, 25 ± 2 °C of temperature, and 300 μmol m^−2^s^−1^ of photon flux density. When plants reached the V8 development stage, which is the development of the eighth node and represents the presence of the seventh fully developed trifoliate leaf [23], we initiated the conduction of the different irrigation regimes.

Plants were submitted to water deficit for eight, six, four, and two days, in two experiments. The soil water potentials obtained were −0.96, −0.38, −0.07, and −0.02 MPa, for the first experiment and −3.09, −1.38, −0.69, and −0.14 MPa, for the second experiment. After these days without irrigation, xenobiotic and antioxidant agents were applied (Table 1). Accordingly, to treatments, some plants received xenobiotic, others received only antioxidant agents, and in the remainder were applied concomitantly xenobiotic and antioxidant agents, which is how it would be applied in the field.

The experiment comprised sixteen treatments with three replicates laid out in a completely randomized design.

### 2.2. Procedures

The products were applied using a spray bar pressurized with CO_2_, with a volume of 150 L ha^−1^ and flat jet tip deflector (TT11002). One hour after application were initialized the physiological measures, while biochemical collections were made 48 h after application.

Soil samples were collected during all the experiments to obtain the water content of the soil and substrate mixture. Water retention capacity was determined at the end of the experiment when preserved structure samples were collected through volumetric cylinders. These samples were then saturated with water in porous plate funnels and chamber, then subjected to increasing tensions of 0.01, 1, 6, 10, 100, and 300 kPa. Retained water content at higher potentials was obtained by 10 deformed soil samples placed in metal capsules, and potential was determined using the WP4-T Dewpoint Potentiameter (Decagon Devices, Inc., Pullman, WA, USA). Moisture results, as a function of water potential, were adjusted to the model of van Genuchten (Supplementary Materials data) in the Soil Water Retention Curve (SWRC) software, version 3.00 beta [24].

### 2.3. Physiological Parameters

#### 2.3.1. Plant Water Status and Stomatal Conductance

Plant water status was evaluated through the leaf water potential and relative water content (RWC). Leaf water potential was evaluated in the sixth fully developed trifoliate leaf using a Scholander pressure chamber (Soilmoisture Equipment Corp., Model 3115, Santa Barbara, CA, USA). Plant RWC was determined in the fifth fully developed trifoliate leaf according to Cavalcanti et al. [25]. Was collected 30 leaf discs of one centimeter and weighted; after were put in Petri dishes with distilled water during 7 h, at 25 °C under 300 umol m^2^ s^−1^ light intensity. Then, were weighted to obtain turgid weight. Leaf discs were dried in an air circulation oven, at 60 °C until constant weight, and there weighted again. Relative water content was obtained by: [(fresh leaf disc weight—dry leaf disc weight)/(turgid leaf disc weight—dry leaf disc weight) *100]. Stomatal conductance was evaluated using an AP4 leaf porometer (Delta-T Devices, Burwell, Cambridge, UK).

#### 2.3.2. Leaf Temperature and Photosynthetic Pigments

Leaf temperature was evaluated in the seventh fully developed trifoliate leaf through a digital infrared thermometer (RayTemp, model 8, Electronic Temperature Instruments LTD., Worthing, UK), with an emissivity of 0.95. Total chlorophyll content was evaluated in the fourth fully developed trifoliate leaf by a chlorophyll meter (Falker, model ClorofiLog, Porto Alegre, Brasil). Values were expressed by the Falker chlorophyll index, which is a relative value of the chlorophyll content and is based on correlations of absorbance and reflectance.

#### 2.3.3. Photosystem II Quantum Yield and Chlorophyll Fluorescence

Chlorophyll fluorescence and photosystem II (PSII) quantum yield were evaluated in the fourth fully developed trifoliate leaf with a fluorometer (FluorPen, model FP-100, Photon Systems Instruments, Drásov, Czech Republic).

### 2.4. Biochemical Analysis

#### 2.4.1. Enzymatic Extraction and Protein

Eight and ninth fully developed trifoliate leaf were collected, macerated, and homogenized (use of a buffer in a volume of four times the fresh mass) in potassium phosphate buffer solution (pH 6.8), centrifuged at 3000 rpm and 250 of G-force for 10 min (centrifuge Centri-Bio, model 80-2B, São Paulo, Brasil). The supernatant (extract) was collected and stored at −80 °C for further analysis.

The sample protein was determined according to the Bradford method [26]. Were added 300 µL of the extract and 3 mL of Bradford in tubes. These tubes were incubated in the dark for 10 min. After that, the absorbance was read in a spectrophotometer (Kasuaki, model IL-227, São Paulo, Brasil) at 595 nm. Protein content was calculated based on an equation obtained from the standard curve of albumin.

#### 2.4.2. Reactive Oxygen Species (ROS) and Oxidative Damage

Hydrogen peroxide (H_2_O_2_) production was determined according to the methodology of Loreto and Velikova [27]. Analysis was carried out in duplicates, on an Elisa plate, being added to each well 50 µL of enzymatic extract, 50 µL of 1M potassium iodide, and 100 µL of potassium phosphate buffer (pH 7.0). Absorbance was determined on a spectrophotometer (Elisa reader, Synergy H1, Biotek, Winooski, VT, USA) at 390 nm. H_2_O_2_ concentrations of samples were estimated by multiplying the absorbance by the correction factor of 0.54.

In situ detection of H_2_O_2_ was determined according to Thordal-Christensen [28]. The seventh fully developed trifoliate leaf was collected, placed in test tubes containing 10 mM buffer solution (pH 5.8) of MES and DAB (3.3′-Diaminobenzidine tetrahydrochloride hydrate). Tubes were incubated in light of 300 µmol m^2^ s^−1^ for 12 h. After this period, the solution was removed from tubes, and 95% alcohol was added. Tubes with alcohol were put in a water bath at 100 °C for 10 min to remove chlorophyll. Then trifoliate leaves were removed and photographed.

Superoxide (O_2_^•−^) production was estimated by Chaitanya and Naithani [29]. Analysis was conducted in duplicate, adding 100 µL of the extract to a mixture containing 100 mM of sodium phosphate buffer (pH 7.2), 1 mM of sodium diethyldithiocarbamate, and 0.25 mM nitro-tetrazolium blue. Absorbance was measured in a spectrophotometer (Kasuaki, model IL-227, São Paulo, Brasil), at 540 nm, for three min and the concentration determined by subtracting the final absorbance (at three min) from the initial absorbance (at 0 s).

Membrane damage (MD) was estimated in the sixth fully developed trifoliate leaf, from the electrolyte loss, by Cavalcanti et al. [25] methodology. From these trifoliate, were removed 20 leaf discs of one centimeter, placed in falcon tubes containing 20 mL of deionized water. These were kept under stirring for 12 h at 25 °C. After that was evaluated the hydraulic water conductivity with a digital conductivity meter. Then, tubes were placed in a water bath during 1 h, at 95 °C, for the release of all electrolytes. After that time, tubes were removed, cooled and hydraulic conductivity reading was performed again. Membrane damage was calculated by dividing the initial reading by the final reading and multiplied by 100 for transformation into a percentage.

The content of malondialdehyde (MDA), which estimates lipid peroxidation, was determined according to the methodology of Heath and Packer [30], who quantifies the substances reactive to thiobarbituric acid (TBARS) present in the tissue. Were placed in a tube 400 µL of enzymatic extract, 100 µL of trichloroacetic acid (10%), and 500 µL of thiobarbituric acid (0.67%). This tube was incubated in a water bath at 100 °C for 15 min, cooled and centrifuged at 2000 rpm, and a G-force of 250 (centrifuge Centri-Bio, model 80-2B, São Paulo, Brasil) for 15 min. The absorbance was measured in a spectrophotometer (Kasuaki, model IL-227, São Paulo, Brasil), at 535 nm, and the MDA content was calculated from the equation generated by the standard curve with MDA.

#### 2.4.3. Antioxidant Enzymes Activity and Antioxidant Non-Enzymatic

Superoxide dismutase (SOD) activity was measured by the capacity of SOD to photochemically reduce the p-nitrotetrazolium blue (NTB), according to Del Longo et al. [31]. Analysis were performed in duplicates, with 40 μL of enzyme extract added in 960 μL of a mixture containing 50 mM of potassium phosphate buffer (pH 7.8), 13 of mM methionine, 75 μM of nitro-tetrazolium blue, 0.1 mM of ethylenediamine tetra-acetic acid (EDTA) and 2 µM of riboflavin. This homogenate was kept for 10 min at 25 °C incubated under light (lamp of 15W). After this period, a spectrophotometer (Pró-análise, model UV-1600, Porto Alegre, Brasil) reading was performed at 560 nm, which recorded the production of formazan blue, resulting from the photorecovery of nitro-tetrazolium blue [32]. One of the whites was kept with the samples incubated in the light (white of the light), and the other kept in the dark, for 10 min. The value obtained for the samples was subtracted from the value found in the subtraction of the reading of the dark white by the reading of the white of the light. Thus, a unit of SOD is defined as the amount of enzyme required to inhibit NBT photoreduction by 50% [33].

Catalase (CAT) activity was determined by adapting the method proposed by Cakmak and Marschner [34] based on the H_2_O_2_ decomposition rate. Duplicates were made, adding 50 μL of enzymatic extract in 1950 μL of a mixture containing 50 mM of potassium phosphate buffer (pH 6.8) and 20 mM of H_2_O_2_. The reaction was measured on a spectrophotometer (Pró-análise, model UV-1600, Porto Alegre, Brasil), at 240 nm for one minute at 25 °C. CAT activity was calculated by subtracting the final absorbance (1 min) from the initial absorbance (0 s) and divided by the extinction coefficient of 36 M cm^−1^ [35].

Ascorbate peroxidase (APX) activity was measured by the Nakano and Asada [36] methodology, using an extinction coefficient of 2.8 mM cm^−1^. Was added 25 μL of enzyme extract in a mixture containing 50 mM potassium phosphate buffer (pH 6.8), 1 mM H_2_O_2_, and 0.8 mM of ascorbate. The readings were measured in a spectrophotometer (Pró-análise, model UV-1600, Porto Alegre, Brasil), at 290 nm by the rate of ascorbic oxidation in one min, at 25 °C. APX activity was calculated by subtracting the final absorbance (1 min) from the initial absorbance (0 s) and divided by the extinction coefficient of 2.8 mM cm^−1^.

Non-protein thiols content, an indirect glutathione determination, was estimated by the method adapted from Griffith [37]. They were placed into a tube 500 μL of enzymatic extract, with 500 μL of 10% TCA; placed to centrifuge (centrifuge Centri-Bio, model 80-2B, São Paulo, Brasil) for 20 min at 2000 rpm and a G-force of 250. 800 μL of the supernatant was removed and reacted with 80 μL of 5,5′-ditiobis-2-nitrobenzoic acid (DTNB). The absorbance was determined in a spectrophotometer (Kasuaki, model IL-227, São Paulo, Brasil), at 412 nm, and the content of non-protein thiols calculated based on the equation obtained by the curve generated from cysteine standards.

### 2.5. Statistical Analysis

The normality of residuals of the data were verified by the Shapiro−Wilk test. The data that needed transformation was highlighted in tables and figures. Some data needed to be transformed, and the type of transformation used is highlighted in the tables and figures. Data were submitted to analysis of variance (ANOVA), regression equations were performed, and when necessary, means are compared by the Tukey test at 5% error probability. Only significant results found in the analysis of variance are shown in the work. The analysis was performed in the R software using ExpDes.pt package [38]. In the Statistica software, multivariate techniques were performed using factor analysis, Principal Component Analysis, and correlations techniques. Variables were standardized for these analyses.

## 3. Results

### 3.1. Plant Water Status and Stomatal Conductance

**Figure 1:**
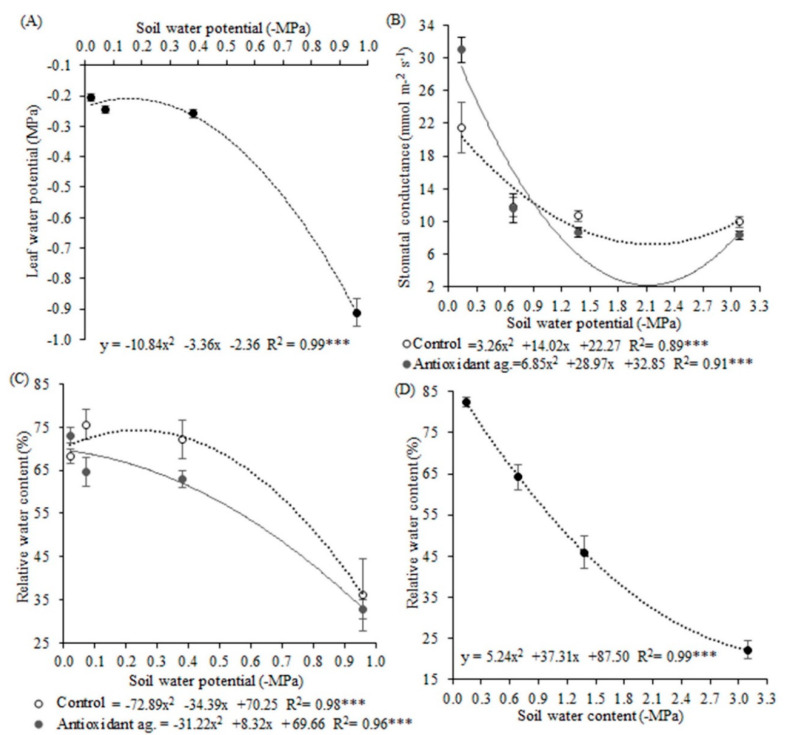
Leaf water potential (**A**); stomatal conductance (**B**); relative water content in the first experiment (**C**) and the second experiment (**D**) of soybean plants under different soil water potentials and antioxidant agent application. Note: asterisks indicate error probability as * = 10%, ** = 5%, and *** = 1%, respectively. Vertical bars represent the standard error of the samples. Analysis of leaf water potential and stomatal conductance were made with transformed data. Leaf water potential data were transformed by cubic root, and stomatal conductance data were transformed by a logarithmic function.

Leaf water potential presented a response with quadratic behavior, and 99% of the variation was explained by the soil water potential (Figure 1A). The leaf water potential had variations of −0.21 MPa, for the soil water potential of −0.02 MPa, up to −0.91 for the soil water potential of −0.96 MPa. Leaf water potential in the lowest soil water potential (−0.96 MPa) was 75% lower than the other potentials, which did not themselves differ significantly.

The quadratic response was also observed for RWC in both experiments. In the first experiment, 98% of the variation in the control was explained by soil water potential, and 96% of the variation in the antioxidant agent treatment was explained by soil water potential. Plants had 50% lower RWC after eight days of deficit water than plants six, four, and two days under this stress in the control and antioxidant agent treatments (Figure 1C). The antioxidant agent application was not shown to be effective for improving plant water status. In the second experiment, RWC varied between 22 to 82% and this 88% of this variation was explained by soil water potential (Figure 1D).

More gas exchanges were observed for the plants under the highest soil water potential (−0.14 MPa), which presented stomatal conductance values 60% higher than plants in the other soil water potentials (Figure 1B). In this highest potential, the plants treated with the antioxidant agent presented 0.3-fold increases in stomatal conductance in comparison to the control.

### 3.2. Leaf Temperature and Photosynthetic Pigments

**Figure 2:**
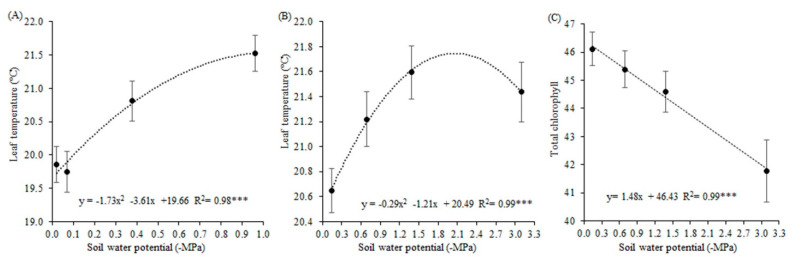
Leaf temperature in the first experiment (**A**) and the second experiment (**B**); Total chlorophyll (**C**) of soybean plants under different soil water potentials and xenobiotic and antioxidant agent applications. Note: asterisks indicate error probability as * = 10%, ** = 5%, and *** = 1%, respectively. Vertical bars represent the standard error of the samples.

In the first experiment, the soybean plants under eight days of water restriction presented the highest leaf temperature (21.5 °C), 0.7 °C higher than plants under six days of water deficit, and, on average, 1.7 °C higher than plants without irrigation for two and four days (Figure 2A). Leaf temperature showed a quadratic increase behavior, contrary to RWC and conductance because plants under higher soil water potentials presented decreased temperature. The soil water treatments were responsible for 98% of the variation in temperature. The interaction between the xenobiotic and antioxidant agent applications showed that the higher temperature was found in the control, and the leaf temperatures were decreased by the application of the products (Table 2). In the second experiment, the leaf temperature presented a quadratic behavior, with 99% of the variation explained by the soil water potential (Figure 2B) The higher leaf temperature was found by the plants with −3.09 and −1.38 MPa of soil water potential. The interaction between xenobiotic and antioxidant agents showed lower temperatures in the control (Table 3). When the antioxidant agent was applied, the temperature was increased if the xenobiotic was applied, and when the xenobiotic was applied, the temperature was increased if the antioxidant agent was applied concomitantly.

In the first experiment, total chlorophyll was reduced by more than 5% by xenobiotic and antioxidant agent application compared to the control (Table 3). Total chlorophyll was adjusted by a decrescent linear regression, with 99% of the variation explained by the soil water potential. Soybean plants were negatively influenced by deficit water stress, with reductions of almost 10% in these pigments from the highest to the lowest soil water potential (Figure 2C). For each −1 MPa of soil water potential, total chlorophyll decreased 1.48.

### 3.3. Photosystem II Quantum Yield and Chlorophyll Fluorescence

**Figure 3:**
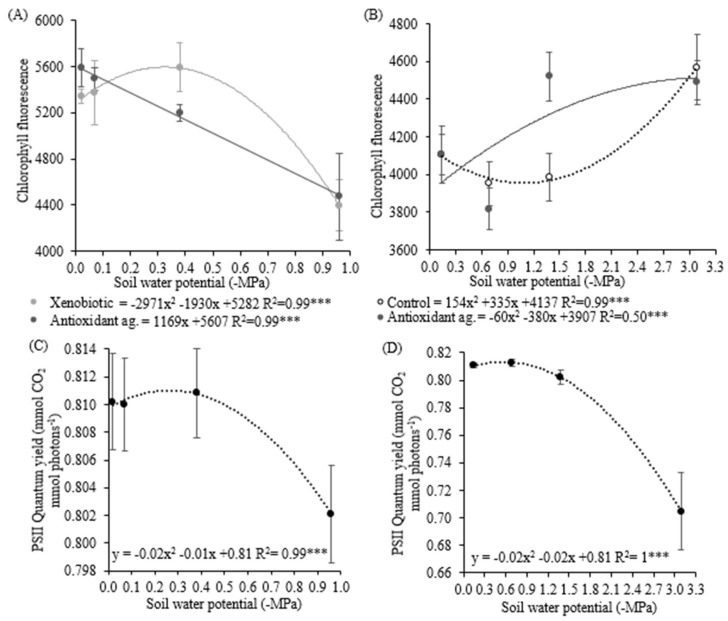
Chlorophyll fluorescence in the first experiment (**A**) and the second experiment (**B**), quantum yield of photosystem II in the first experiment (**C**), and the second experiment (**D**) in soybean plants under different soil water potentials, and xenobiotic and antioxidant agent application. Note: asterisks indicate error probability as * = 10%, ** = 5%, and *** = 1%, respectively. Vertical bars represent the standard error of the samples.

PSII quantum yield decreased in plants exposed to the lowest soil water potential, in both experiments (Figure 3C,D), in a quadratic behavior, with variations explained 99% and 100% by soil water potential, respectively, in the first and second experiment. Higher PSII quantum yield was observed in control plants (Table 4). In the first experiment, chlorophyll fluorescence was reduced in plants submitted to xenobiotic and antioxidant agent applications at the lowest soil water potentials, with models adjusted 99% (Figure 3A). The application of the xenobiotic increased chlorophyll fluorescence by almost 5% (Table 5). The interaction table shows that fungicide application increases chlorophyll fluorescence in plants that had not received antioxidant agent applications in soil water potentials of −0.38, −0.07, and −0.02 MPa, and with an antioxidant agent in soil water potential of −0.96 MPa (Table 6). Antioxidant agent application increases chlorophyll fluorescence in plants without xenobiotic in soil water potentials of −0.38, −0.07, and −0.02 MPa and in the soil water potential of −0.96 in plants with the xenobiotic. In the second experiment, control plants and plants treated with antioxidant agents presented higher fluorescence in the lowest soil water potentials (Figure 3B). Antioxidant agent-treated plants showed more chlorophyll fluorescence, compared to control, only in soil water potential of −1.38 MPa. Soil water potential explained 50% and 100% of the fluorescence variation, respectively, for the control and antioxidant agent.

### 3.4. ROS and Oxidative Damages

**Figure 4:**
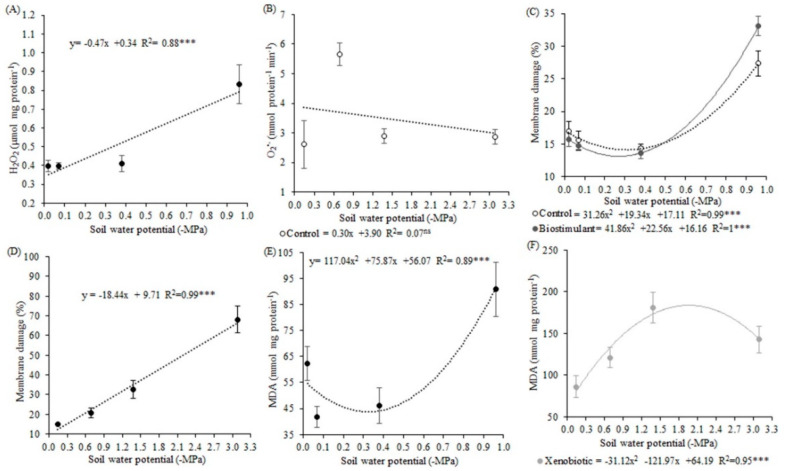
Hydrogen peroxide (**A**) and superoxide production (**B**), membrane damage in the first experiment (**C**) and the second experiment (**D**), malondialdehyde content in the first experiment (**E**) and the second experiment (**F**) in soybean plants under different soil water potentials and xenobiotic and antioxidant agent application. Note: asterisks indicate error probability as * = 10%, ** = 5%, and *** = 1%, respectively. Vertical bars represent the standard error of the samples. Analysis of hydrogen peroxide and membrane damage in the first experiment was made with transformed data. Hydrogen peroxide data were transformed by square root, and membrane damage data were transformed by a logarithmic function.

Hydrogen peroxide showed significance only in the first experiment when a higher concentration of H_2_O_2_ was observed for plants under drought for eight days, in soil water potential of −0.96 MPa (Figure 4A). For each unit of soil water potential, H_2_O_2_ production increased by 0.47 µmol mg protein^−1^, with 88% of the variation explained by the soil water potential. Antioxidant agent application reduced the production of H_2_O_2_ by 0.16 µmol mg protein^−1^, which represents more than 25% of reduction (Table 7). In the first experiment, O_2_^•−^ was reduced when only one of the products was applied, either an antioxidant agent without xenobiotic or xenobiotic without an antioxidant agent (Table 8). Control plants under −0.69 MPa presented almost 50% higher O_2_^•−^ concentration than plants under the other potentials (Figure 4B). The interaction table showed differences in soil water potential of −0.69 MPa (Table 9). In this potential, plants without an antioxidant agent and with the xenobiotic showed less O_2_^•−^ production compared to plants that did not receive the xenobiotic.

**Figure 5:**
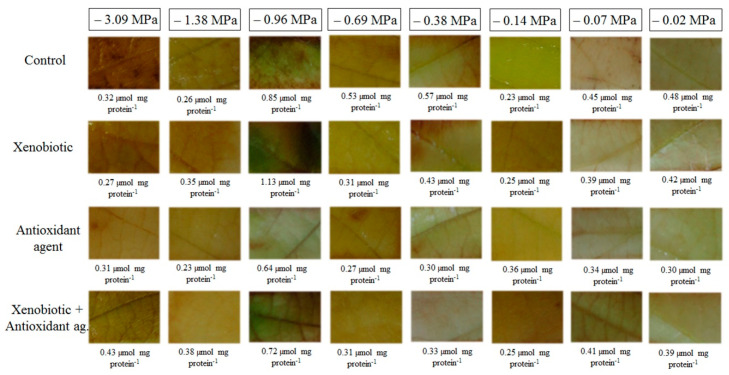
In situ detection of hydrogen peroxide in soybean plants under different soil water potentials and xenobiotic and antioxidant agent applications.

In situ detection of H_2_O_2_ showed that the production of this ROS was higher in conditions of more severe stress (lower soil water potentials) (Figure 5). Leaves of the plants grown in soil water potential of −0.96 MPa showed the highest number of brown spots, indicating more sites with the H_2_O_2_ presence. Plants treated with a xenobiotic, alone or in combination with the antioxidant agent, also presented higher spots, demonstrating a higher concentration of H_2_O_2_ in plant cells, while the plants treated only with the antioxidant agent showed a marked reduction in the spots, indicating a lower production of H_2_O_2_.

Membrane damage (MD) presented increasing quadratic behavior in the first experiment (Figure 4C). The soil water potential explained 99% of the variation in the control and 100% of the variation in the antioxidant agent treatments. In the lowest soil water potential, membrane damage was 50% higher, compared to the other potentials, and more damage was observed to the antioxidant agent-treated plants. In the second experiment, the highest MD was observed in soil water potential of −3.09 MPa, which was 66% higher than the other potentials, which did not differ from each other (Figure 4D). The increase was linear, and 99% of the variation was explained by the soil water potential.

In the first experiment, the malondialdehyde content (MDA) of soybean plants under deficit water for eight days was 45% higher when compared to the MDA of plants for six, four, and two days under deficit (Figure 4E). The regression was quadratic, with 89% of the variation explained by soil water potential. In the second experiment, xenobiotic application increased lipid peroxidation in plants submitted to −1.38 and −3.09 MPa (Figure 4F). According to the regression model, the soil water potentials explained 95% of the variations observed. The interaction table between applications showed higher MDA when the antioxidant agent was applied concomitantly with the xenobiotic (Table 10).

### 3.5. Antioxidants

**Figure 6:**
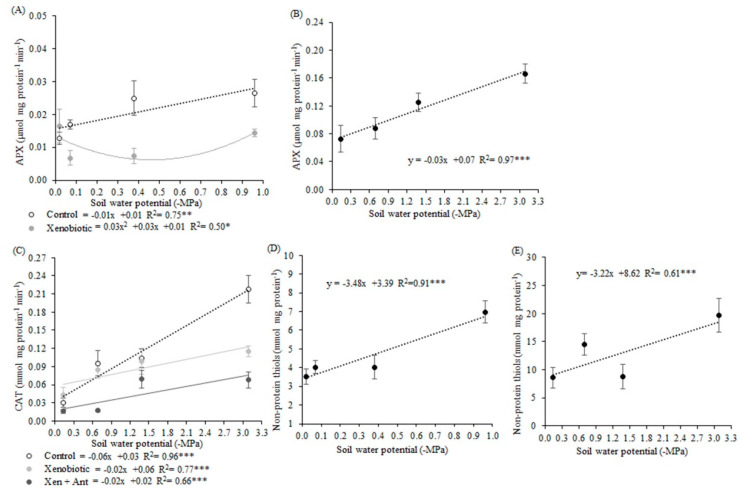
Ascorbate peroxidase activity in the first experiment (**A**) and the second experiment (**B**). Catalase activity in the second experiment (**C**). Non-protein thiols in the first experiment (**D**) and the second experiment (**E**) in soybean plants under different soil water potentials and xenobiotic and antioxidant agent applications. Note: asterisks indicate error probability as * = 10%, ** = 5%, and *** = 1%, respectively. Vertical bars represent the standard error of the samples.

In the first experiment, the APX activity was reduced by the xenobiotic in all soil water potentials, except for −0.02 MPa (Figure 6A). In the second experiment, APX was increased in lower soil water potentials, in a linear behavior, with 97% of the variation explained by soil water potentials (Figure 6B). In the second experiment, CAT activity increased linearly in the control, xenobiotic, and xenobiotic + antioxidant agent plants (Figure 6C). The interaction table shows that when plants did not receive a xenobiotic application, CAT activity was higher without an antioxidant agent in −3.09, −1.38, and −0.69 MPa (Table 11). This enzyme activity was increased with the xenobiotic and without the antioxidant agent in −3.09 and −0.69 MPa plants.

All antioxidant enzyme activities were reduced by antioxidant agent applications (Table 12). SOD was reduced by 15% and 25% in the first and second experiments, respectively. APX was reduced by 37% and 48% in the first and second experiments, respectively, and CAT activity was reduced by 57% in the first experiment.

The highest values of non-protein thiols were observed in the treatments with low water availability (Figure 6D,E). Plants that received antioxidant agent application in the first experiment show a 30% decrease in thiols content (Table 12).

### 3.6. Principal Component Analysis (PCA)

The PCA was performed to found the variables that more explained the variations found in the interactions studied in this work. Therefore, the analysis of the experiment I resulted in 2 factors with eigenvalues higher than one, which resulted in a cumulative 77.218% of the cumulative variance of all analyzed variables (Table 13). Factor 1 is composed of leaf water potential, leaf temperature, chlorophyll fluorescence, H_2_O_2_, O_2_^•−^, lipid peroxidation, and non-protein thiols; Factor 2: chlorophyll, RWC, PSII quantum yield, and SOD (Table 14).

**Figure 7:**
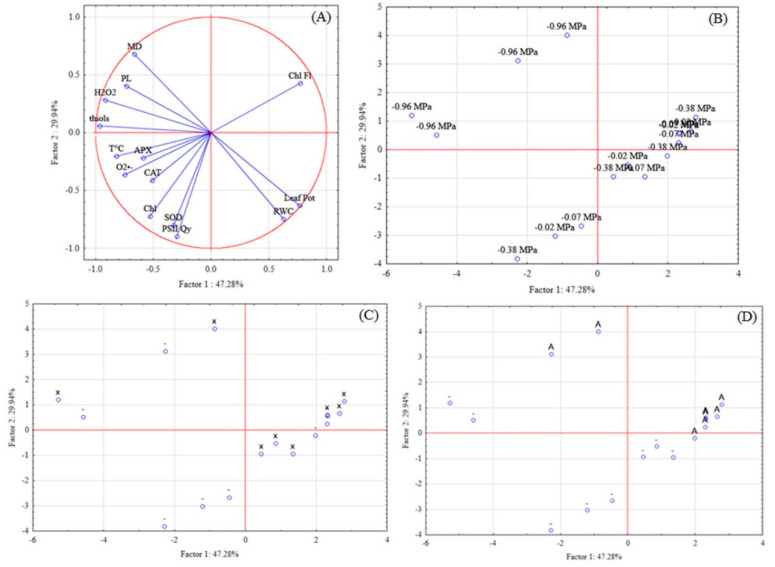
Projection of the variables (**A**) and cases—soil water potential (**B**), xenobiotic (**C**), and antioxidant agent (**D**) on the factor-plane of physiological and biochemical variables of soybean plants in the experiment I. * Abbreviations—Chl: chlorophyll; Leaf Pot: leaf water potential; RWC: relative water content; T°C: leaf temperature; Chl Fl: chlorophyll fluorescence; PSII Qy: photosystem II quantum yield; H_2_O_2_: hydrogen peroxide; O_2_^•−^: superoxide; MD: membrane damage; PL: lipid peroxidation; CAT: catalase; APX: ascorbate peroxidase; SOD: superoxide dismutase; x: xenobiotic, A: antioxidant agent; - without application.

Projection of the variables on the factor-plane of the first experiment was performed using the factors 1 and 2. The first factor explained 47.279% and the second 29.939% (Table 13). The variables that more explained the variation were that localized more closely to the graph border, being the RWC, leaf water potential, H_2_O_2_, membrane damage, and thiols (Figure 7).

The projection of the variables on the factor-plane was performed using factors 1 and 2. The first factor explained 47.279% and the second 29.939% (Table 13). The variables that more explained the variation were that localized more closely to the graph border, being the RWC, leaf water potential, H_2_O_2_, membrane damage, and thiols (Figure 7).

Cases projection on the factor plane shows the relation of the lowest soil water potential, xenobiotic and antioxidant agent application with the ROS production (H_2_O_2_), oxidative damages (MD and PL), and antioxidant defense (thiols).

Still, it can be seen the correlation between the variables, and their interdependence: leaf water potential with the RWC; SOD and PSII quantum yield; leaf temperature with O_2_^•−^ production and APX and CAT activities; H_2_O_2_ with MD, PL, and thiols. Negative relations with plant water status and ROS production, antioxidant system, and damages were found, showing that as the lower water content of the plant, the more impaired will be the oxidative metabolism.

**Figure 8:**
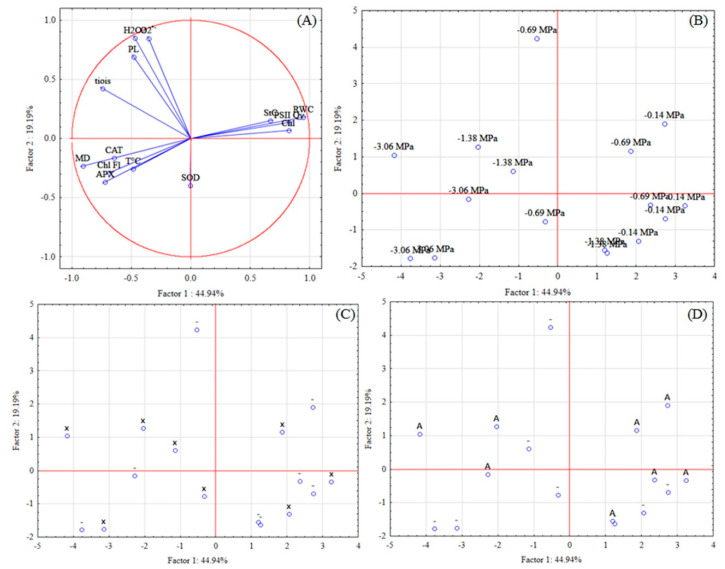
Projection of the variables (**A**) and cases—soil water potential (**B**), xenobiotic (**C**), and antioxidant agent (**D**) on the factor-plane of physiological and biochemical variables of soybean plants in experiment II. * Abbreviations—Chl: chlorophyll; StC: stomatal conductance; RWC: relative water content; T°C: leaf temperature; Chl Fl: chlorophyll fluorescence; PSII Qy: photosystem II quantum yield; H_2_O_2_: hydrogen peroxide; O_2_^•−^: superoxide; MD: membrane damage; PL: lipid peroxidation; CAT: catalase; APX: ascorbate peroxidase; SOD: superoxide dismutase; x: xenobiotic, A: antioxidant agent; - without application.

The projection of the variables on the factor-plane of experiment II was performed also using the two first factors (1 and 2). The first factor explained 44.939% and the second 19.189%. The variables that more explained the variation were localized closer to the graph border: RWC, H_2_O_2_, O_2_^•−^, and MD (Figure 8).

Regarding the cases projection on the factor plane it was observed in the same four-square of the lower soil water potential (−3.09 MPa), the variables indicatives of stress, such as chlorophyll fluorescence, leaf temperature, MD, lipid peroxidation; reactive oxygen species, H_2_O_2_ and O_2_^•−^, and variables of the antioxidant defense system, such as CAT, APX, SOD, and thiols.

It is possible to observe a clear response between the stress combination, water deficit, and xenobiotic, with the oxidative metabolism, as with the mitigation stress with the antioxidant agent. In contrast, higher soil water potentials (control) provided higher plants RWC, stomatal conductance, PSII quantum yield, and chlorophyll content.

H_2_O_2_, O_2_^•−^, PL, and thiols are highly correlated, also as leaf temperature, chlorophyll fluorescence, membrane damage, and CAT and APX activities.

## 4. Discussion

Studies have shown that there is a relationship between decreasing water potential with water deficit conditions [39,40,41]. Literature uses leaf water potential values of −1.2 to −1.5 MPa as critical and indicative of water deficit stress for corn plants, which has a C4 metabolism and is, therefore, more resistant to water deficit [42]. In this work, which was developed in a controlled environment, values of −0.9 MPa indicated more severe deficit water stress for soybean plants. This value may be suggested as a stress indicator for the soybean crop.

Water deficit and concurrent xenobiotic application comprised a new stress situation, in agreement with [11], not simply based on adding the isolated effects of deficit water to the isolated effects of xenobiotic. Other studies had shown positive results when stresses were combined, compared to isolated stresses [43,44,45,46]. This shows the complexity involved in different stress conditions, isolated or in combinations.

Plants exposed to deficit water stress had reduced their water status, leaf water potential, RWC, and stomatal conductance. These physiological changes are commonly observed and corroborate with [47]. The decrease of the stomatal conductance reduced the gas exchanges, which may have contributed to the decrease in CO_2_ absorption, which also favored the increase in the leaf temperature of these plants. Under low soil water availability, plants closed their stomata to prevent water loss by transpiration, maintain cell turgor, and regulate CO_2_ absorption [46,48]. According to these authors, the heat and water that would be removed from the leaves by evaporative cooling stayed in the cells, increasing leaf temperature.

This increasing temperature may have contributed to the degradation of photosynthetic pigments, or even alteration in the biosynthesis of these pigments because they were reduced. According to [49], these cited factors are considered the main cause of pigment reduction in stress situations. Reduced chlorophyll content also may be due to lower photosynthesis of plants, and oxidative stress. [50] also observed reductions in the photosynthetic activity of soybean plants when under soil water potential of −0.026 MPa. Photosynthetic pigments were also reduced by xenobiotic application, which can cause less photon absorption by these molecules and consequently diminish the plant’s ability to absorb light.

Quantum yield was also affected by water deficit, and the plants under this stress for eight days (−3.09 MPa) had a quantum yield of less than 0.75 mmol CO_2_ mmol photons^−1^, which is considered a critical limit. Below this limit, plants may suffer damage to PSII [51]. The reduction in the quantum yield of PSII results from the decrease in the efficiency with which the PSII reaction centers capture the energy [52]. Chlorophyll fluorescence is an evaluation of plant stress, then the increased chlorophyll fluorescence in xenobiotic treated plants indicated changes in photosynthetic activity [53], an indication of greater photoinhibition and consequent physical damage to PSII.

Increased ROS production, mainly H_2_O_2_, was observed in plants under water deficit, a result that is already well known in the literature [54,55,56,57]. This species accumulation may be due to reductions in liquid photosynthesis, triggered by the CO_2_ limited entry, due to the stomatal closure [54]. Moreover, thermal stress due to deficit causes disturbances in electron flow and destabilization of the energy reactions, which leads to ROS accumulation [48]. Increased production of these molecules in cells can cause an imbalance in the antioxidant system, which may result in the generation of oxidative stress [58].

The proportion of this increase may vary in each situation, depending on the stress intensity and duration, plant development stage, genetics, and even the evaluated tissue [59]. Thus, in this work, the highest concentration of H_2_O_2_ was not observed for the lowest soil water potential (−3.09 MPa, in the second experiment), except for plants submitted to −0.96 MPa. A possible reason for this is that ROS, such as H_2_O_2_, are considered as signaling molecules of stress despite being toxic molecules at a certain concentration in cells [60]. Thus, alterations in plant metabolism, such as increased production of H_2_O_2_ molecules, were triggered under water deficit and may have signaled this stressful situation, and thus the plant induced defense mechanisms.

The different behavior of the observed ROS species can be due to the anticipated production of O_2_^•−^ at lower soil water potential (−0.69 MPa). This is because O_2_^•−^ is usually the first ROS generated, being produced by oxygen reduction during electron transport along the electron transport chain [61]. In the O_2_^•−^ dismutation, there is a reduction of two electrons of oxygen, which is a determinant factor in H_2_O_2_ and hydroxyl radical formation [61]. Hydroxyl radical is the most toxic and reactive molecule formed by the Fenton reaction, which occurs in the presence of transition metals, like copper and iron [41,60,61].

We observed that a combined water deficit and fungicide application increased H_2_O_2_ production by 25%, which is very considerable. Changes in H_2_O_2_ levels in cells can act as indicators of the membrane structural integrity of stress-exposed plants [62] because membranes are often the first target of abiotic stresses [9]. In this sense, the greatest membrane damage observed in plants of the lower soil water potentials may have been due to higher ROS production. According to [63] under stress conditions, ROS production increase results in electrolyte leakage, which leads to MD increases.

The MDA content is another indication of membrane damage that was increased in plants under water deficit. MDA is the last product of lipid peroxidation that occurs in the membranes [64]. The effect of increasing leaf temperature caused by lower water availability increased membrane fluidity, which is related to the rupture and peroxidation of the lipids constituent of these structures, corroborating [9]. This damage was potentialized by xenobiotic application in the second experiment.

As H_2_O_2_ increased in combined stresses, the MDA content also increased. Because higher MDA contents represent oxidative damages that may be caused by ROS, fungicide application in plants submitted to very low soil water potentials may lead to the generation of oxidative stress. This H_2_O_2_ content estimated at 0.83 mmol mg protein^−1^ min^−1^ at the potential −0.96 MPa, may, therefore, be suggested as a borderline. This value could be considered to separate what would be a basal level of H_2_O_2_, which is essential and positive for the plant, from a level that could be considered critical and capable of causing damage, and which was therefore signaled and neutralized by antioxidant machinery.

Antioxidant agent application resulted in the lowest ROS production, and this stress mitigation by this biostimulant would be due to its composition. It contains folcysteine, which is formed by folic acid and cysteine. The sulfur element needs to be reduced to sulfate and cysteine to be assimilated by the plant. There is a need for electron donation among the steps for this reduction, and glutathione is one of the possible donors [48]. Thus, glutathione, an important antioxidant, is related to cysteine and can act in the stress attenuation when the antioxidant agent is applied. According to the projection of the variables and cases, in PCA, glutathione can be suggested as the main compound of the product as a stress mitigator.

Thermal stress due to water deficit may impair plant metabolism because of its effect on protein and enzyme stability [48]. Activation of defense mechanisms such as antioxidant enzymes, like induction of APX activity and non-protein thiols, predominantly glutathione (GSH), was observed with increases in temperature (low soil water potentials). These defense mechanisms are a result of increased respiratory activity. GSH represent the non-enzymatic antioxidant compounds and is formed by the enzyme glutathione reductase, which uses NADPH to reduce GSH-oxidized glutathione that acts by directly reducing ROS [65].

Defense antioxidant mechanisms are considered important adaptive strategies to minimize oxidative damage and were activated to combat the ROS generated by stress situations [60,61,66]. Thus, higher thiol contents that were found in the lower soil water potentials showed the increased antioxidant activity to protect the plant from water deficit. Incremental CAT activity was observed in lower soil water potentials, with a linear response similar to H_2_O_2_ production, because this enzyme acts on the H_2_O_2_ defense route [67].

As observed for CAT, the activity of APX was also increased in deficit water stress for the control plants. APX is considered an active defense line in the neutralization of excessive production and possible negative effects of H_2_O_2_ [67]. According to [10], enzymes such as CAT and glutathione peroxidase, for example, are induced in water deficit conditions. Other defense routes can be triggered for combined stresses, such as glutathione reductase (GR) and glutathione-S-transferase.

Antioxidant agent application reduced SOD, CAT, and APX activities in the different soil water potentials. Another hypothesis to be considered is that the mitigation of oxidative stress, visualized by the lower ROS production in plants treated with the antioxidant agent, may have resulted due to the presence of folcysteine on its composition. Cysteine residues can react with O_2_^•−^ and with H_2_O_2_, with constant reaction rates varying by 7 × 105 and 1-2, respectively, for these two species [60,68,69,70].

ROS species interact with several signaling pathways, and signals are transmitted through proteins, with cysteine being the target of these molecules [68]. Cysteine may be in several states of oxidation because it contains the sulfur atom with many electrons, causing cysteine residues to be important modification sites [71]. Signal transduction sensors can be unraveled by these modified proteins that contain cysteine [72]. The cysteine, as well as glutamate, glycine, and phenylalanine, are considered signaling amino acids, which in small doses can increase the activity of antioxidant enzymes [73].

Thus, the antioxidant agent may have been essential for stress signaling by the ROS, which may have contributed to an increase in the activity of the antioxidant machinery and a reduction in the production and harmful effects of ROS. However, studies such as that of [74] only recognize H_2_O_2_ as a secondary messenger, considering O_2_^•−^ only as a precursor of H_2_O_2_ and not a direct participant in signaling. It could also be supposed by the multivariate analysis that the defense mechanisms, like the catalase enzyme and the glutathione (thiols) can be activated by the antioxidant agent, which minimized the damage. The ROS may be the result of combined stresses, such as ROS or as signaling molecules that allow the early perception of stress.

Biostimulants, like the antioxidant agent used in this study, ameliorate the uptake and efficiency of nutrients and also increase the tolerance to abiotic stress [19,75]. This tolerance was probably obtained from modifications in antioxidant properties, such as enzyme activities [76]. Biostimulants improve the antioxidant defense capacity of plants, which helps in stress tolerance. However, it is noticeable that there is a need for studies that indicate which mechanisms are responsible for this, a fact also observed by [77].

Although folcysteine is not the only component of the antioxidant agent used in this study, it is the one with the highest amount. The interactions between the components of this product are complex and potentiate the folcysteine action. There is no way to indicate which component is responsible for the observed effects but to state that the effect comes from this formulation, which contributes to the mobility of folcysteine, in the case of nitrogen and phosphorus [48]. According to the same authors, zinc, magnesium, and potassium acting in the activation of the enzymes contributes to the antioxidant system being metal enzymatic cofactors [48]. In addition, potassium also regulates osmotic potential, and iron and manganese are known for redox reactions and electron transfer [48]. Therefore, the sum of each isolated effect led to the potentiation of the effects and resulted in the ability to mitigate the effects of water deficit and xenobiotic stresses.

It is important to highlight that results, according to the literature that already exists, that plants under stress closure stomata, reducing gas exchanges and consequently photosynthesis. These alterations lead to mitochondrial respiration changes and the generation of ROS in photosynthetic electron transport [60]. These toxic molecules can cause chemical damage to DNA, protein damage, impaired cellular effects, and gene expression changes.

With the antioxidant agent application, some of the antioxidant defenses were activated, neutralizing the toxic effects of ROS, and maintaining a basal level able to signal the stress, and preventing harmful effects, and also modulating cell expansion throughout the stiffness and relaxation of the cell wall [67]. Amino acids, nutrients, hormones, and humic acids containing in the biostimulant composition probably triggered metabolic alterations in the plants, that quite changed the oxidation responses to stress, but maybe had not yet reached the physiological level.

## 5. Conclusions

**Figure 9:**
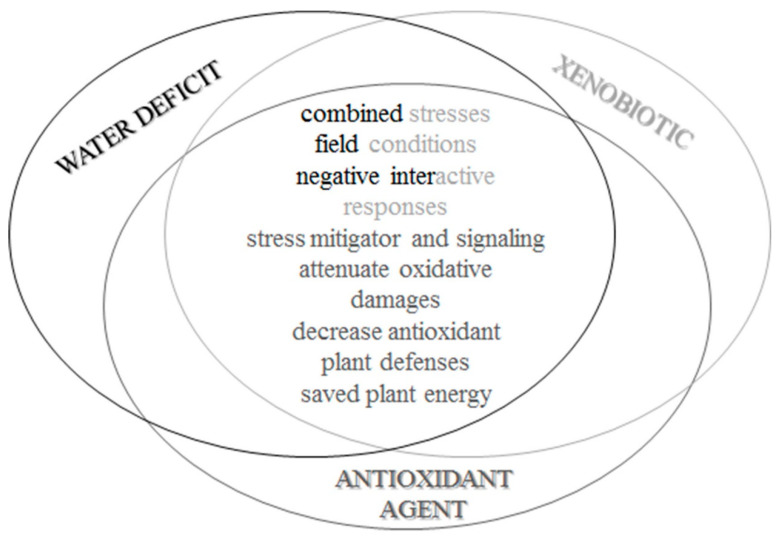
Conclusion figure about results found in this article, showing the interactions of the combination of water deficit and xenobiotic stress and the antioxidant agent mitigating in soybean plants.

We concluded that soybean plants under water deficit for six and eight days in a controlled environment were subjected to oxidative stress, by the increased ROS production, cellular and molecular damages, and induction of defense antioxidant metabolism. In addition, the plants, presented physiological changes, such as the reduction of gas exchange, water status, and photosynthetic efficiency. The xenobiotic application also caused changes in oxidative metabolism. Negative effects were more pronounced when the xenobiotic application occurred in low soil water availability. This indicates that responses triggered from concurrent stresses are different from those triggered by isolated stresses, being observed negative interactive responses for these stressors combinations, and the most deleterious effect probably caused by water deficit. Antioxidant agent application was favorable for attenuating the effects of water deficit and xenobiotic application in soybean plants (Figure 9). We suggest the potential of −0.9 MPa for the water potential of the leaf and soil as a limit for the application of xenobiotics, or if it is necessary to apply in more severe conditions, an alternative is the addition of the antioxidant agent that showed a potential for reducing cellular and oxidative damages in soybean plants.

## Figures and Tables

**Table 1:** Xenobiotic and antioxidant agent compositions.

Xenobiotic	Ingredients	Quantity	Antioxidant Agent	Ingredients	Quantity
FOX^®^		g L^−1^	Foltron Plus^®^		g L^−1^
	trifloxystrobin	150		humic acids	8
	prothioconazole	175			**%**
	inerts	775		total amoniacal nitrogen	10
				phosphorus	20
				potassium	5
					**ppm**
				magnesium	100
				boron	80
				copper	50
				iron	500
				manganese	100
				molybdenum	2
				zinc	500
				folcysteine	2750
				gibberellins	30

Note: ppm: parts per million.

**Table 2:** Leaf temperature (°C) in soybean plants submitted to xenobiotic and antioxidant agent applications.

Experiment I	Leaf Temperature		Experiment II	Leaf Temperature	
Antioxidant Agent	Xenobiotic		Antioxidant Agent	Xenobiotic	
	Without	With		Without	With
Without	22.00 ± 0.22 aA	20.07 ± 0.28 bA	Without	20.64 ± 0.15 bB	22.23 ± 0.17 aA
With	19.98 ± 0.24 aB	19.89 ± 0.23 aA	With	21.03 ± 0.13 aA	21.01 ± 0.17 bA

Note: Different lowercase and uppercase letters indicate a significant difference in the line and the column, respectively, at a 5% probability of error, by the Tukey test. The standard error of the samples is shown by the ± values.

**Table 3:** Total chlorophyll in soybean plants submitted to xenobiotic and antioxidant agent applications.

Experiment I	Chlorophyll	Experiment II	Chlorophyll
**Control**	49.44 ± 0.62 A	**Control**	48.78 ± 0.89 A
**Antioxidant ag.**	45.41 ± 0.64 B	**Xenobiotic**	46.07 ± 0.44 B

Note: Different uppercase letters indicate a significant difference in the column, at a 5% probability of error, by the Tukey test. The standard error of the samples is shown by the ± values.

**Table 4:** PSII quantum yield (mmol CO_2_ mmol photons^−1^) in soybean plants submitted to xenobiotic and antioxidant agent applications.

Experiment I	PSII Quantum Yield	
Antioxidant Agent	Xenobiotic	
	Without	With
Without	0.825 ± 0.002 aA	0.806 ± 0.002 bA
With	0.804 ± 0.002 aB	0.798 ± 0.002 bB

Note: Different lowercase and uppercase letters indicate a significant difference in the line and the column, respectively, at a 5% probability of error, by the Tukey test. The standard error of the samples is shown by the ± values.

**Table 5:** Chlorophyll fluorescence in soybean plants submitted to the xenobiotic application.

Experiment II	Chlorophyll Fluorescence
Control	4081 ± 81 B
Xenobiotic	4305 ± 79 A

Note: Different uppercase letters indicate a significant difference in the column, at a 5% probability of error, by the Tukey test. ± represent samples standard error.

**Table 6:** Chlorophyll fluorescence in soybean plants submitted to different soil water potentials and xenobiotic and antioxidant agent applications.

Chlorophyll Fluorescence			
Soil Water Potential (MPa)	Antioxidant Agent	Xenobiotic	
		Without	With
−0.96	Without	4239 ± 163 aA	4396 ± 221 aB
	With	4472 ± 373 bA	5662 ± 201 aA
−0.38	Without	4168 ± 132 bB	5593 ± 212 aA
	With	5199 ± 69 aA	5538 ± 92 aA
−0.07	Without	4098 ± 45 bB	5373 ± 275 aA
	With	5494 ± 125 aA	5273 ± 96 aA
−0.02	Without	4332 ± 142 bB	5345 ± 59 aA
	With	5591 ± 111 aA	5545 ± 162 aA

Note: Different lowercase and uppercase letters indicate a significant difference in the line and the column, respectively, at a 5% probability of error, by the Tukey test. The standard error of the samples is shown by the ± values.

**Table 7:** Hydrogen peroxide (µmol mg protein^−1^) in soybean plants submitted to antioxidant agent application.

Experiment I	H_2_O_2_
Control	0.59 ± 0.07 A
Antioxidant ag.	0.43 ± 0.04 B

Note: Different uppercase letters indicate a significant difference in the column, at a 5% probability of error, by the Tukey test. The standard error of the samples is shown by the ± values. The analysis was made with square root transformed data.

**Table 8:** Superoxide (mmol protein^−1^ min^−1^) in soybean plants submitted to xenobiotic and antioxidant agent applications.

O_2_^•−^	Experiment I	
Antioxidant Agent	Xenobiotic	
	Without	With
Without	2.05 ± 0.16 aA	1.66 ± 0.24 bA
With	0.89 ± 0.05 bB	1.26 ± 0.04 aA

Note: Different lowercase and uppercase letters indicate a significant difference in the line and the column, respectively, at a 5% probability of error, by the Tukey test. The standard error of the samples is shown by the ± values. The analysis was made with transformed data by cubic root.

**Table 9:** Superoxide (mmol protein^−1^ min^−1^) in soybean plants submitted to different soil water potentials and xenobiotic and antioxidant agent applications.

O_2_^•−^			
Soil Water Potential (MPa)	Antioxidant Agent	Xenobiotic	
		Without	With
−3.09	Without	2.86 ± 0.25 aA	3.19 ± 0.50 aA
	With	3.45 ± 0.51 aA	4.03 ± 0.28 aA
−1.38	Without	2.89 ± 0.25 aA	3.94 ± 0.91 aA
	With	2.41 ± 0.47 aA	3.69 ± 0.39 aA
−0.69	Without	5.66 ± 0.37 aA	3.44 ± 0.68 bA
	With	2.61 ± 0.34 aB	3.28 ± 0.26 aA
−0.14	Without	2.61 ± 0.81 aA	3.14 ± 0.74 aA
	With	3.65 ± 0.28 aA	2.77 ± 0.38 aA

Note: Different lowercase and uppercase letters indicate a significant difference in the line and the column, respectively, at a 5% probability of error, by the Tukey test. The standard error of the samples is shown by the ± values.

**Table 10:** Malondialdehyde content (mmol mg protein^−1^) in soybean plants submitted to different soil water potentials and xenobiotic and antioxidant agent applications.

Experiment II	MDA	
Antioxidant Agent	Xenobiotic	
	Without	With
Without	115.85 ± 10.66 aA	113.71 ± 10.50 aB
With	109.14 ± 9.80 bA	151.94 ± 15.66 aA

Nore: Different lowercase and uppercase letters indicate a significant difference in the line and the column, respectively, at a 5% probability of error, by the Tukey test. The standard error of the samples is shown by the ± values.

**Table 11:** Catalase (mmol mg protein^−1^ min^−1^) activity in soybean plants submitted to different soil water potentials and xenobiotic and antioxidant applications.

CAT			
Soil Water Potential (MPa)	Antioxidant Agent	Xenobiotic	
		Without	With
−3.09	Without	0.217 ± 0.023 aA	0.115 ± 0.009 bA
	With	0.055 ± 0.005 aB	0.067 ± 0.013 aB
−1.38	Without	0.103 ± 0.015 aA	0.098 ± 0.021 aA
	With	0.036 ± 0.004 aB	0.069 ± 0.014 aA
−0.69	Without	0.095 ± 0.020 aA	0.084 ±0.014 aA
	With	0.043 ± 0.002 aB	0.017 ± 0.002 aB
−0.14	Without	0.030 ± 0.008 aB	0.043 ± 0.012 aA
	With	0.072 ± 0.004 aA	0.016 ± 0.003 bA

Note: Different lowercase and uppercase letters indicate a significant difference in the line and the column, respectively, at a 5% probability of error, by the Tukey test. The standard error of the samples is shown by the ± values. Analysis of catalase activity was made with data transformed by a log function.

**Table 12:** Superoxide dismutase activity (SOD unit mg protein^−1^), catalase activity (mmol mg protein^−1^ min^−1^), ascorbate peroxidase activity (µmol mg protein^−1^ min^−1^), and non-protein thiols content (mmol mg protein^−1^) in soybean plants submitted to antioxidant agent applications.

	Experiment I				Experiment II	
	SOD	CAT	APX	Thiols	SOD	APX
Control	5.11 ± 0.27 A	0.068 ± 0.009 A	0.019 ± 0.002 A	5.53 ± 0.48 A	4.17 ± 0.21 A	0.149 ± 0.010 A
Antioxidant Agent	4.35 ± 0.19 B	0.029 ± 0.003 B	0.012 ± 0.002 B	3.74 ± 0.34 B	3.12 ± 0.17 B	0.077 ± 0.011 B

Note: Different uppercase letters indicate a significant difference in the column, at a 5% probability of error, by the Tukey test. The standard error of the samples is shown by the ± values.

**Table 13:** Explained, total, and cumulative variance in each factor of the PCA for analyzed variables of soybean plants submitted to water deficit, xenobiotic, and antioxidant agent applications.

	Experiment I			Experiment II		
	Variance			Variance		
Factor	Explained	Total (%)	Cumulative (%)	Explained	Total (%)	Cumulative (%)
1	6.619	47.279	47.279	6.291	44.939	44.939
2	4.191	29.939	77.218	2.686	19.189	64.127
3	-	-	-	1.622	11.589	75.717

**Table 14:** Factor loadings of each variable of the two experiments.

	Experiment I		Experiment II		
Variables/Factors	Factor Loadings		Factor Loadings		
	1	2	1	2	3
Leaf Pot	0.764	-	-	-	-
StC	-	-	-	-	-
RWC	-	0.746	0.946	-	-
T°C	−0.821	-	-	-	-
Chl	-	−0.723	0.822	-	-
Chl Fl	0.768	-	-	-	-
PSII Qy	-	−0.895	0.827	-	-
O_2_*^•−^*	0.747	-	-	0.844	-
H_2_O_2_	−0.914	-	-	0.846	-
MD	-	-	−0.906	-	-
PL	−0.734	-	-	-	-
SOD	-	−0.797	-	-	0.824
CAT	-	-	-	-	-
APX	-	-	−0.72	-	-
thiols	−0.966	-	−0.741	-	-

Note: Abbreviations—Leaf Pot: leaf water potential; StC: stomatal conductance; RWC: relative water content; T°C: leaf temperature; Chl: chlorophyll; Chl Fl: chlorophyll fluorescence; PSII Qy: photosystem II quantum yield; H_2_O_2_: hydrogen peroxide; O_2_^•−^: superoxide; MD: membrane damage; PL: lipid peroxidation; CAT: catalase; APX: ascorbate peroxidase; SOD: superoxide dismutase.

## Supplementary Materials

The following are available online at https://www.mdpi.com/2079-7737/9/9/266/s1, Figure S1: Soil retention characteristic curve.

## References

[1] Eldakak M., Milad S.I.M., Nawar A.I., Rohila J.S. (2013). Proteomics: A biotechnology tool for crop improvement. Front. Plant Sci..

[2] Habibi D. (2014). Evaluation of antioxidant enzymes activity in canola under salt stress. MAGNT Res. Rep..

[3] Farnese F.S., Menezes-Silva P.E., Gusman G.S., Oliveira J.A. (2016). When bad guys become good ones: The key role of reactive oxygen species and nitric oxide in the plant responses to abiotic stress. Front. Plant Sci..

[4] Ma X., Wang G., Zhao W., Yang M., Ma N., Kong F., Dong X., Meng Q. (2017). SlCOR413IM1: A novel cold-regulation gene from tomato, enhances drought stress tolerance in tobacco. J. Plant Physiol..

[5] Beruski G.C., Del Ponte E.M., Pereira A.B., Gleason M.L., Câmara G.M.S., Araújo Júnior I.P., Sentelhas P.C. (2020). Performance and profitability of rain-based thresholds for timing fungicide applications in soybean rust control. Plant Dis..

[6] Venancio W.S., Rodrigues M.A.T., Begliomini E., de Souza N.L. (2003). Physiological effects of strobilurin fungicides on plants. Publ. UEPG.

[7] Islam F., Ali B., Wang J., Farooq M.A., Gill R.A., Ali S., Wang D., Zhou W. (2016). Combined herbicide and saline stress differentially modulates hormonal regulation and antioxidant defense system in Oryza sativa cultivars. Plant Physiol. Biochem..

[8] Zandalinas S.I., Balfagón D., Arbona V., Gómez-Cadenas A. (2017). Modulation of antioxidant defense system is associated with combined drought and heat stress tolerance in citrus. Front. Plant Sci..

[9] Farooq M., Wahid A., Kobayashi N., Fujita D., Basra S.M.A. (2009). Plant drought stress: Effects, mechanisms and management. Agron. Sustain. Dev..

[10] Pandey P., Ramegowda V., Senthil-Kumar M. (2015). Shared and unique responses of plants to multiple individual stresses and stress combinations: Physiological and molecular mechanisms. Front. Plant Sci..

[11] Mittler R. (2006). Abiotic stress, the field environment and stress combination. Trends Plant Sci..

[12] Li W., Zhang C., Lu Q., Wen X., Lu C. (2011). The combined effect of salt stress and heat shock on proteome profiling in Suaeda salsa. J. Plant Physiol..

[13] Katam R., Shokri S., Murthy N., Singh S.K., Suravajhala P., Khan M.N., Bahmani M., Sakata K., Reddy K.R. (2020). Proteomics, physiologicalm and biochemical analysis of cross tolerance mechanisms in response to heat and water stresses in soybean. PLoS ONE.

[14] Jumrani K., Bhatia V.S. (2018). Impact of combined stress of high temperature and water deficit on growth and seed yield of soybean. Physiol. Mol. Biol. Plants.

[15] Wang L., Dong S., Liu L., Ma Y., Li S., Zu W. (2018). Transcriptome profiling reveals PEG-simulated drought, heat and combined stress response mechanisms in soybean. Comput. Biol. Chem..

[16] Shen X., Dong Z., Chen Y. (2015). Drought and UV-B radiation effect on photosynthesis and antioxidant parameters in soybean and maize. Acta Physiol. Plant..

[17] Bashir W., Anwar S., Zhao Q., Hussain I., Xie F. (2019). Interactive effect of drought and cadmium stress on soybean root morphology and gene expression. Ecotoxicol. Environ. Saf..

[18] Bilal S., Shahzad R., Imran M., Jan R., Min K., Lee I. (2020). Synergistic association of endophytic fungi enhances Glycine max L. resilience to combined abiotic stresses: Heavy metals, high temperature and drought stress. Ind. Crops Prod..

[19] du Jardin P. (2015). Plant biostimulants: Definition, concept, main categories and regulation. Sci. Hortic. (Amsterdam).

[20] Yakhin O.I., Lubyanov A.A., Yakhin I.A., Brown P.H. (2017). Biostimulants in plant science: A global perspective. Front. Plant Sci..

[21] Ugena L., Hýlová A., Podlešáková K., Humplík J.F., Doležal K., De Diego N., Spíchal L. (2018). Characterization of biostimulant mode of action using novel multi-trait high-throughput screening of Arabidopsis germination and rosette growth. Front. Plant Sci..

[22] Kauffman G.L., Kneivel D.P., Watschke T.L. (2007). Effects of a biostimulant on the heat tolerance associated with photosynthetic capacity, membrane thermostability, and polyphenol production of perennial ryegrass. Crop Sci..

[23] Fehr W.R., Caviness C.E. (1977). Stages of Soybean Development.

[24] Dourado-neto D., Nielsen D.R., Hopmans J.W., Reichardt K., Bacchi O.O.S. (2000). Software to model soil water retention curves (SWRC, version 2.00). Sci. Agric..

[25] Cavalcanti F.R., Oliveira J.T.A., Martins-Miranda A.S., Viégas R.A., Silveira J.A.G. (2004). Superoxide dismutase, catalase and peroxidase activities do not confer protection against oxidative damage in salt-stressed cowpea leaves. New Phytol..

[26] Bradford M.M. (1976). A rapid and sensitive method for the quantitation of microgram quantities of protein utilizing the principle of protein-dye binding. Anal. Biochem..

[27] Loreto F., Velikova V. (2001). Isoprene produced by leaves protects the photosynthetic apparatus against ozone damage, quenches ozone products, and reduces lipid peroxidation of cellular membranes. Plant Physiol..

[28] Thordal-Christensen H., Zhang Z., Wei Y., Collinge D.B. (1997). Subcellular localization of H_2_O_2_ in plants. H_2_O_2_ accumulation in papillae and hypersensitive response during the barley-powdery midew interaction. Plant J..

[29] Chaitanya K.S.K., Naithani S.C. (1994). Role of superoxide, lipid peroxidation and superoxide dismutase in membrane perturbation during loss of viability in seeds of Shorea robusta Gaertn.f. New Phytol..

[30] Heath R.L., Packer L. (1968). Photoperoxidation in isolated chloroplasts. Arch. Biochem. Biophys..

[31] Del Longo O.T., Gonzalez C.A., Pastori G.M., Trippi V.S. (1993). Antioxidant defences under hyperoxygenic and hyperosmotic conditions in leaves of two lines of maize with differential sensitivity to drought. Plant Cell Physiol..

[32] Giannopolitis C.N., Ries S.K. (1977). Superoxide Dismutases—I. Occurrence in higer plants. Plant Physiol..

[33] Beauchamp C., Fridovich I. (1971). Superoxide Dismutase: Improved assays and an assay applicable to acrylamide gels. Anal. Biochem..

[34] Cakmak I., Marschner H. (1992). Magnesium deficiency and high light intensity enhance activities of superoxide dismutase, ascorbate peroxidase, and glutathione reductase in bean leaves. Plant Physiol..

[35] Anderson M.D., Prasad T.K., Stewart C.R. (1995). Changes in isozyme profiles of catalase, peroxidase, and glutathione reductase during acclimation to chilling in mesocotyls of maize seedlings. Plant Physiol..

[36] Nakano Y., Asada K. (1980). Spinach chloroplasts scavenge hydrogen peroxide on illumination. Plant Cell Physiol..

[37] Griffith O.W. (1980). Determination of glutathione and glutathione disulfide using glutathione reductase and 2-vinylpyridine. Anal. Biochem..

[38] R Core Team (2019). R: A Language and Environment for Statistical Computing.

[39] Makbul S., Saruhan Güler N., Durmuş N., Güven S. (2011). Changes in anatomical and physiological parameters of soybean under drought stress. Turk. J. Botany.

[40] Khan M.S., Karim M.A., Abullah-Al-Mahmud, Parveen S., Bazzaz M.M., Hossain M.A. (2015). Plant water relations and proline accumulations in soybean under salt and water stress environment. J. Plant Sci..

[41] Choudhury F.K., Rivero R.M., Blumwald E., Mittler R. (2017). Reactive oxygen species, abiotic stress and stress combination. Plant J..

[42] Bergonci J.I., Bergamaschi H., Berlato M.A., Santos A.O. (2000). Potencial da água na folha como um indicador de déficit hídrico em milho. Pesqui. Agropecu. Bras..

[43] Pérez-López U., Miranda-Apodaca J., Muñoz-Rueda A., Mena-Petite A. (2013). Lettuce production and antioxidant capacity are differentially modified by salt stress and light intensity under ambient and elevated CO_2_. J. Plant Physiol..

[44] Iyer N.J., Tang Y., Mahalingam R. (2013). Physiological, biochemical and molecular responses to a combination of drought and ozone in Medicago truncatula. Plant Cell Environ..

[45] Rivero R.M., Mestre T.C., Mittler R., Rubio F., Garcia-Sanchez F., Martinez V. (2014). The combined effect of salinity and heat reveals a specific physiological, biochemical and molecular response in tomato plants. Plant Cell Environ..

[46] Zandalinas S.I., Mittler R., Balfagón D., Arbona V., Gómez-Cadenas A. (2018). Plant adaptations to the combination of drought and high temperatures. Physiol. Plant..

[47] Javadi T., Rohollahi D., Ghaderi N., Nazari F. (2017). Mitigating the adverse effects of drought stress on the morpho-physiological traits and anti-oxidative enzyme activities of Prunus avium through β-amino butyric acid drenching. Sci. Hortic. (Amsterdam).

[48] Taiz L., Zeiger E., Max I., Angus M. (2017). Fisiologia e Desenvolvimento Vegetal.

[49] Aghaie P., Tafreshi S.A., Ebrahimi M.A., Haerinasab M. (2018). Tolerance evaluation and clustering of fourteen tomato cultivars grown under mild and severe drought conditions. Sci. Hortic. (Amsterdam).

[50] Chavarria G., Durigon M.R., Klein V., Kleber H. (2015). Restrição fotossintética de plantas de soja sob variação de disponibilidade hídrica. Cienc. Rural.

[51] Suassuna J.F., de Melo A.S., Costa F.S., Fernandes P.D., Ferreira R.S., da S Sousa M.S. (2011). Eficiência fotoquímica e produtividade de frutos de meloeiro cultivado sob diferentes lâminas de irrigação. Semin. Ciências Agrárias.

[52] Zhang J., Liu J., Yang C., Du S., Yang W. (2016). Photosynthetic performance of soybean plants to water de fi cit under high and low light intensity. South African J. Bot..

[53] Baker N.R., Rosenqvist E. (2004). Applications of chlorophyll fluorescence can improve crop production strategies: An examination of future possibilities. J. Exp. Bot..

[54] Das A., Eldakak M., Paudel B., Kim D.-W., Hemmati H., Basu C., Rohila J.S. (2016). Leaf proteome analysis reveals prospective drought and heat stress response mechanisms in soybean. Biomed Res. Int..

[55] Wang H., Yang L., Li Y., Hou J., Huang J., Liang W. (2016). Involvement of ABA- and H_2_O_2_-dependent cytosolic glucose-6-phosphate dehydrogenase in maintaining redox homeostasis in soybean roots under drought stress. Plant Physiol. Biochem..

[56] Xing X.-h., Fang C.-w., Li L., Jiang H.-q., Zhou Q., Jiang H.-d., Wang S.-h. (2016). Improved drought tolerance by α-naphthaleneacetic acid-induced ROS accumulation in two soybean cultivars. J. Integr. Agric..

[57] Nahar S., Vemireddy L.R., Sahoo L., Tanti B. (2018). Antioxidant protection mechanisms reveal significant response in drought-induced oxidative stress in some traditional rice of Assam, India. Rice Sci..

[58] Cruz de Carvalho M.H. (2008). Drought stress and reactive oxygen species: Production, scavenging and signaling. Plant Signal. Behav..

[59] Schneider J.R., Caverzan A., Chavarria G. (2019). Water deficit stress, ROS involvement, and plant performance. Arch. Agron. Soil Sci..

[60] Mittler R. (2017). ROS Are Good. Trends Plant Sci..

[61] Gill S.S., Tuteja N. (2010). Reactive oxygen species and antioxidant machinery in abiotic stress tolerance in crop plants. Plant Physiol. Biochem..

[62] Shahid M., Ahmed B., Zaidi A., Khan M.S. (2018). Toxicity of fungicides to Pisum sativum: A study of oxidative damage, growth suppression, cellular. RSC Adv..

[63] Foyer C.H., Leiandais M., Kunert K.J. (1994). Photooxidative stress in plants. Physiol. Plant..

[64] Sahin U., Ekinci M., Ors S., Turan M., Yildiz S., Yildirim E. (2018). Effects of individual and combined effects of salinity and drought on physiological, nutritional and biochemical properties of cabbage (Brassica oleracea var. capitata). Sci. Hortic. (Amsterdam).

[65] Noctor G., Foyer C.H. (1998). Ascorbate and glutathione: Keeping active oxygen under control. Annu. Rev. Plant Physiol. Plant Mol. Biol..

[66] Guler N.S., Pehlivan N. (2016). Exogenous low-dose hydrogen peroxide enhances drought tolerance of soybean (Glycine max L.) through inducing antioxidant system. Acta Biol. Hung..

[67] Mhamdi A., Breusegem F.V. (2018). Reactive oxygen species in plant development. Development.

[68] Akter S., Huang J., Waszczak C., Jacques S., Gevaert K., Breusegem F.V., Messens J. (2015). Cysteines under ROS attack in plants: A proteomics view. J. Exp. Bot..

[69] Winterbourn C.C. (2015). Are free radicals involved in thiol-based redox signaling?. Free Radic. Biol. Med..

[70] Smirnoff N., Arnaud D. (2019). Hydrogen peroxide metabolism and functions in plants. New Phytol..

[71] Davies M.J. (2005). The oxidative environment and protein damage. Biochim. Biophys. Acta.

[72] Couturier J., Chibani K., Jacquot J., Rouhier N. (2013). Cysteine-based redox regulation and signaling in plants. Front. Plant Sci..

[73] Teixeira W.F., Fagan E.B., Soares L.H., Umburanas R.C., Reichardt K., Neto D.D. (2017). Foliar and seed application of amino acids affects the antioxidant metabolism of the soybean crop. Front. Plant Sci..

[74] Forman H.J., Maiorino M., Ursini F. (2010). Signaling functions of reactive oxygen species. Biochemistry.

[75] Rouphael Y., Spíchal L., Panzarová K., Casa R., Colla G. (2018). High-throughput plant phenotyping for developing novel biostimulants: From lab to field or from field to lab?. Front. Plant Sci..

[76] Drobek M., Frac M., Cybulska J. (2019). Plant biostimulants: Importance of the quality and yield of horticultural crops and the improvement of plant tolerance to abiotic stress—A review. Agronomy.

[77] Savy D., Brostaux Y., Cozzolino V., Delaplace P., du Jardin P., Piccolo A. (2020). Quantitative structure-activity relationship of humic-like biostimulants derived from agro-industrial byproducts and energy crops. Front. Plant Sci..

