# Supplementary figures and images for: Soybean Plant Metabolism under Water Deficit and Xenobiotic and Antioxidant Agent Application
Source: Biology (Basel). 2020 Sep 3;9(9):266. doi: 10.3390/biology9090266 (PMC7565094; doi:10.3390/biology9090266)

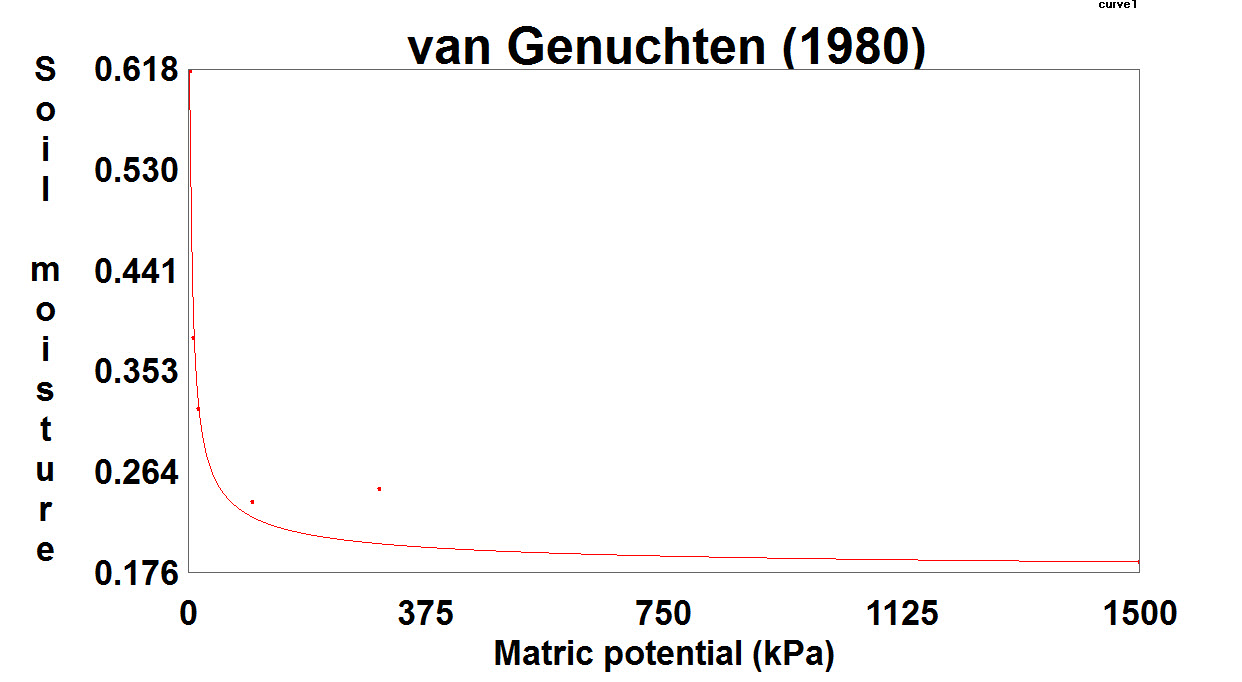

Supplement: Supplementary file 1 [file biology-09-00266-s001.jpg]
